# What Should We Do after the COVID-19 Vaccination? Vaccine-Associated Diseases and Precautionary Measures against Adverse Reactions

**DOI:** 10.3390/vaccines10060866

**Published:** 2022-05-28

**Authors:** Toru Awaya, Masao Moroi, Yoshinari Enomoto, Taeko Kunimasa, Masato Nakamura

**Affiliations:** Department of Cardiovascular Medicine, Toho University Ohashi Medical Center, 2-22-36, Ohashi Meguro-ku, Tokyo 153-8515, Japan; moroi@med.toho-u.ac.jp (M.M.); yenomo1225@oha.toho-u.ac.jp (Y.E.); taesan@live.jp (T.K.); masato@oha.toho-u.ac.jp (M.N.)

**Keywords:** COVID-19 vaccination, cardiovascular diseases, vaccine-associated diseases, adverse reaction, inflammatory cytokine, autoimmunity, lipid nanoparticles, precautionary measures, exercise, taking a bath

## Abstract

COVID-19 vaccines have been used to counteract the global COVID-19 pandemic. While these are effective, adverse reactions have been reported, such as injection-site pain, muscle ache, fever, palpitation, and chest discomfort. The release of inflammatory cytokines, such as interleukin (IL)-6 and IL-1β, is a potential mechanism for post-vaccine side-effects. Chest discomfort after the vaccination, including myocarditis and acute coronary syndrome, is a particularly serious adverse reaction. It is important to be familiar with the differential diagnoses of chest discomfort and organ-specific diseases associated with COVID-19 vaccines as the preparation for booster shots and vaccinations among children aged 5–11 years begins. High-intensity exercise, alcohol, tobacco smoking, and baths promote inflammatory cytokines, such as IL-6, which may exacerbate the adverse reactions after vaccination. Japanese data show that deaths during baths are the most common for several days after mRNA vaccination. Additionally, alcohol and tobacco smoking were identified as predictive factors of lower antibody titers after vaccination. In this review, we aimed to provide a few recommendations to prevent vaccine-associated disease.

## 1. Introduction

In Japan, four COVID-19 vaccines have been approved for public use, namely, the Pfizer-BioNTech (BNT162b2) messenger RNA (mRNA) vaccine, Moderna (mRNA-1273) mRNA vaccine, Oxford/AstraZeneca (ChAdOx1 nCoV-19) adenovirus vectored vaccine, and Novavax (NVXCoV2373) recombinant spike protein nanoparticle vaccine [1,2]. The post-vaccine symptoms often last 1–2 days following the injection [3]. Adverse reactions are more frequently reported in younger individuals, women, individuals who have received the second dose, and individuals with a history of COVID-19 infection [3,4]. The most common systemic reactions, such as muscle aches (69.1%), headaches (48.7%), fever (32.1%), chest discomfort (3.0%), etc., have been reported after the second dose of the BNT162b2 mRNA vaccine [3,4]. The reported rates of serious adverse reactions, including deaths per million doses of mRNA vaccines, are as follows: death was 15, coagulopathy was 14.5, seizure was 9.1, stroke was 6.5, Bells’ palsy was 6.4, anaphylaxis was 5.5, myocarditis was 4.4, acute coronary syndrome (ACS) was 3.7, appendicitis was 1.3, and Guillain-Barré syndrome (GBS) was 1.0 [5]. Yeo et al. reported that the second post-vaccination (60.6%) occurs more often than the first vaccination (39.4%) in the death cases [6].

Inflammatory cytokines release [7,8,9,10], autoimmunity involvement [11,12,13,14,15,16,17,18,19], eosinophil association [20,21,22,23,24,25], and angiotensin-converting enzyme 2 (ACE2) downregulation [26,27] have been suggested as contributing etiologies of post-vaccine adverse reactions. Inflammatory cytokines, including interleukin (IL)-6 and IL-1β, are released due to lipid nanoparticles (LNPs) within the mRNA vaccine [9]. The COVID-19 mRNA vaccine encodes the SARS-CoV-2 spike protein, which triggers IL-1β secretion in macrophages [28]. Not only were immunostimulatory cytokines such as interferon (IFN)-γ released, but inflammatory cytokines were also released, especially following the second vaccination [10].

**Figure 1:**
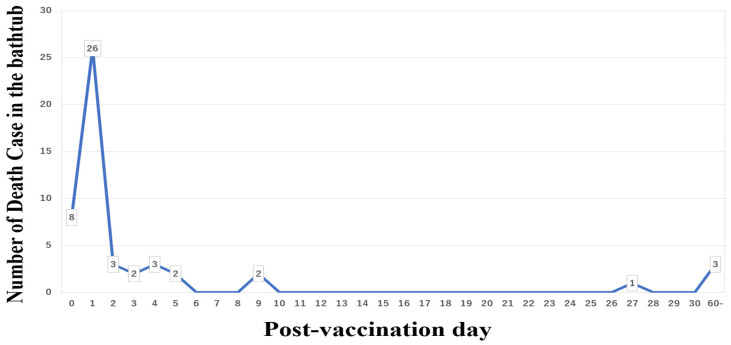
Deaths while sitting in the bathtub after COVID-19 mRNA vaccination.

High-intensity exercise promotes the release of inflammatory cytokines [29]. Drinking alcohol, smoking tobacco, and baths can also increase inflammatory cytokines release [30,31,32]. In Singapore, individuals are advised against strenuous exercise after vaccination [33]. In Japan, deaths while taking a bath have been reported to occur within one week after mRNA vaccination [34,35] (Figure 1).

In this review, we summarized the diseases associated with the COVID-19 vaccines (Table 1) and recommended several precautions to be taken post-vaccination, including limiting high-intensity exercise, alcohol use, tobacco smoking, and baths.

## 2. Organ-Specific Diseases Associated with the COVID-19 Vaccines

### 2.1. Cardiovascular Diseases

**Figure 2:**
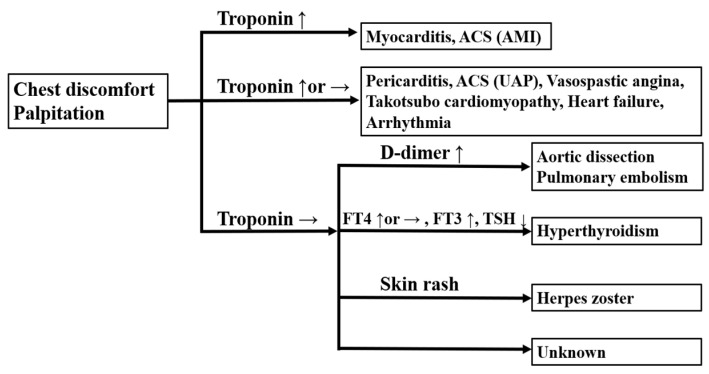
A flowchart of the differential diagnoses of chest discomfort and palpitation after COVID-19 vaccination. ACS: acute coronary syndrome; AMI: acute myocardial infarction; UAP: unstable angina pectoris; FT4: free thyroxine; FT3: free triiodothyronine; TSH: thyroid-stimulating hormone.

Various cardiovascular diseases have been reported to be associated with the COVID-19 vaccine. These include myocarditis and pericarditis [5,20,27,36,37,38,39,40,41], ACS [5,6,7,36], aortic dissection [5,6,34,35], vasospastic angina [36], Takotsubo cardiomyopathy [42], heart failure [5,7,27], arrhythmia [5,14,27,39,43], and pulmonary embolism [5,44] (Table 1). Stark et al. reported the interplay between inflammation cytokines and thrombosis in cardiovascular pathology [45]. The COVID-19 vaccine promotes inflammatory cytokine release [7,8,9,10] and can cause cardiovascular events, including myocarditis (inflammation), ACS (thrombosis), etc. Cardiovascular diseases are the most common causes of death after COVID-19 vaccination [5,6]. Hence, we created a flowchart of the differential diagnoses of chest discomfort and palpitation after COVID-19 vaccination (Figure 2). Myocarditis and pericarditis are reported to be more common in young males after the second vaccination. In male patients aged 12–15 and 16–17 years, the reported incidence is 162.2/million and 93.0/million, respectively [40]. Patients with myocarditis/pericarditis usually present 24–72 h post-vaccination [38]. In contrast, patients with ACS tend to be older in age and typically present 24 h post-vaccination [36]. Oster et al. reported that 98% of post-vaccine myocarditis cases showed an elevated troponin level [38]. Troponin is useful for screening post-vaccine myocarditis, but false negatives are possible, especially within 12 h of the vaccine or a few days later [46]. Electrocardiography and transthoracic echocardiography (TTE) have detected 72% and 17% of the abnormalities associated with post-vaccine myocarditis, respectively [46]. Therefore, diagnosis by multi-modality imaging, including cardiac magnetic resonance imaging and longitudinal strain measured by TTE, is important [37,41]. In cases where a definitive diagnosis is difficult due to the inability to perform multi-modality imaging, detailed follow-up is critical in any cases of suspected myocarditis/pericarditis. NSAIDs, colchicine, and steroid therapy are the standard treatments for myocarditis/pericarditis [27]. In severe cases, steroids may be effective in preventing cytokine release, autoimmunity, and eosinophilic myocarditis [20]. Colchicine, which has an inhibitory effect on the NOD-like receptor family pyrin domain containing 3 (NLRP3) inflammasome, which is associated with IL-1β (inflammatory cytokine) secretion [47], may be also effective for vaccine-associated inflammation [48].

### 2.2. Respiratory Diseases

Asthma attacks [49], diffuse alveolar hemorrhages [50], eosinophilic pneumonia [21], interstitial lung disease [51], and sarcoidosis [52] following COVID-19 vaccination have been reported (Table 1). Although the relationship between the COVID-19 vaccines and asthma attacks and interstitial lung disease is unknown, there have been reports of cardiac arrest after vaccination [34,35,49]. The deaths associated with respiratory disease are the third most common after cardiovascular and cerebrovascular disease [5]. Differentiating these cases from heart failure is important, especially in individuals who exhibit coughing and dyspnea.

### 2.3. Gastroenterological Diseases

Appendicitis [5,53], autoimmune hepatitis (AIH) [11,13], bleeding duodenal ulcer [6], intestinal obstruction/perforation [5], mesenteric ischemia [5], and pancreatitis [54] have been reported post-vaccination (Table 1). Some reported cases of AIH occurred secondary to autoimmune diseases such as primary biliary cholangitis [55,56].

### 2.4. Renal Diseases

Wu et al. reported that minimal change disease, IgA nephropathy, and vasculitis are common in post-vaccine renal disease. Other cases following COVID-19 vaccination include membranous nephropathy relapse, the acute rejection of a kidney transplant, IgG4 nephritis relapse, new-onset renal thrombotic microangiopathy, and scleroderma renal crisis [11,12] (Table 1).

### 2.5. Neurological Diseases

Garg et al. reported many diseases associated with COVID-19 vaccines [57], including acute disseminated encephalomyelitis [58], acute hemorrhagic leukoencephalitis [59], autoimmune encephalitis (AE) [60], Bells’ palsy [5,57], cerebral hemorrhage [61], cerebral infarction [6], cerebral venous sinus thrombosis [62], chronic inflammatory demyelinating polyneuropathy (acute-onset) [63], GBS [5,11,64], multiple sclerosis (MS) [65], myasthenia gravis (MG) [66], neuromyelitis optica spectrum disorder (NMOSD) [16], Parsonage-Turner syndrome (Neuralgic amyotrophy) [67], subarachnoid hemorrhage [6,68], thrombophlebitis [69], and transverse myelitis [70] (Table 1). The second most common cause of death after cardiovascular disease is cerebrovascular disease [5]. IL-6 causes blood-brain barrier dysfunction and enhanced leukocyte transmigration [71], leading to inflammation of the central nervous system. Moreover, IL-6 is also involved in producing anti-aquaporin-4 antibodies, and it has been reported that IL-6 has a higher level in NMOSD [72]. Intracerebral hemorrhages after vaccination due to central venous sinus thrombosis [68], vasculitis [61], and Moyamoya disease with Sjogren disease [73] have been reported. In cases of cerebral hemorrhage after COVID-19 vaccination, thrombosis, vasculitis, and autoimmune diseases should also be considered.

### 2.6. Skin Diseases

Alopecia areata (AA) [74], bullous pemphigoid [75], COVID arm (local injection site reaction) [76,77], eosinophilic cellulitis (Wells syndrome) [22], eosinophilic panniculitis [24], erythema multiforme [78], herpes zoster (skin, oral and facial palsy) [53,57,79,80], leukocytoclastic vasculitis [81], non-episodic angioedema with eosinophilia [23], psoriasis [82], Stevens–Johnson syndrome [83], subacute cutaneous lupus erythematosus [11,84,85], and urticaria [77] have been reported following COVID-19 vaccination (Table 1). AA is an autoimmune disease, and an increase in IFN-γ and inflammatory cytokines, including IL-6 and IL-1β, have been reported [86]. Eosinophilic cellulitis and panniculitis are thought to be type IV hypersensitivity reactions with an increase in IL-4 and IL-5 [24,87]. AA, eosinophilic cellulitis, and eosinophilic panniculitis have been reported not only after COVID-19 vaccination but also after SARS-CoV-2 infection [74,87]. The reactivation of herpes zoster has been reported after mRNA vaccination. The causes are thought to be the dysregulation of T cell function due to vaccine-induced immunomodulation [57].

### 2.7. Endocrine Diseases

Graves’ Disease [88,89], hypophysitis [90], hypothyroidism [88], thyroiditis (painful, silent, and subacute) [88,91], syndrome of inappropriate antidiuresis [92], and Type 1 diabetes mellitus [15] have been reported following COVID-19 vaccination (Table 1). Jafarzadeh et al. reported thyroid dysfunction following COVID-19 vaccination [88], and Yamamoto et al. reported a case of thyroid storm [14]. Therefore, the evaluation of the thyroid hormones after vaccination is important. It is thought that autoimmune diseases, including Graves’ Disease and hypothyroidism, are associated with cross-reactivity. Autoantibodies are produced by the cross-reactivity between thyroid tissue antigen and the SARS-CoV-2 spike proteins produced by mRNA vaccines [93].

### 2.8. Collagen Diseases

Anti-neutrophil cytoplasmic antibody (ANCA)-associated vasculitis [94], antiphospholipid syndrome (APS) [95], dermatomyositis (DM) [96], eosinophilic granulomatosis (EGPA) relapse [25], giant cell arteritis [97], polymyalgia rheumatica [98], rheumatoid arthritis (RA) [11], systemic lupus erythematosus (SLE) [99], and systemic sclerosis (SSc) [100] have been reported after COVID-19 vaccination (Table 1). Autoimmune diseases, including ANCA-associated vasculitis, DM, RA, SLE, and SSc, have been reported [11,94,99,100]. Jinno et al. reported systemic thrombotic events after vaccination in a patient positive for the antiphospholipid antibody. They also suggested that the vaccine may have triggered the onset of APS (second hit) (95).

### 2.9. Hematologic Diseases

Aplastic anemia [101], acquires hemophilia A [102], autoimmune hemolytic anemia [103], hemophagocytic lymphohistiocytosis [28], immune thrombocytopenia [104], and vaccine-induced immune thrombotic thrombocytopenia [105] have been reported after COVID-19 vaccination (Table 1). In each reported case, the main disease was suspected to be related to autoimmunity. Hematologic adverse reactions may not be easily diagnosed, especially if anemia progresses slowly or if the onset of symptoms is slow [103].

### 2.10. Others

Abnormal menstrual cycles (delayed menstruation or increased bleeding or pain) [106], anaphylaxis [44], gout flares [48], lymphadenopathy [107,108], rhabdomyolysis [109], shoulder injuries related to vaccine administration (SIRVA) [109,110], and Vogt–Koyanagi–Harada syndrome [19] have also been reported following COVID-19 vaccination (Table 1). SIRVA is an acute inflammation of the shoulder that causes substantial shoulder pain and a limited range of motion [109,110]. FDG uptake with positron emission tomography imaging, which suggests the inflammation of the deltoid muscle and axillary lymph nodes at the inoculation site, has been reported [107,108].

## 3. Plausible Causes of Post-Vaccine Adverse Reactions

### 3.1. Inflammatory Cytokines

One of the most plausible causes of post-vaccine adverse reactions is the increased release of inflammatory cytokines [7,8,9,10]. Inflammatory cytokines, such as IL-6, IL-1β, etc., are released due to the LNP component of the mRNA vaccines [9]. LNPs are also components of the small interfering RNA therapeutics (Patisiran) [111]. Patisiran increases IL-6 and interferon-inducible protein 10 (IP10) levels after infusion [112]. In practice, patisiran requires premedication before infusion, including dexamethasone, an H1/H2 blocker, and acetaminophen, preventing cytokine release and injection-site reaction [112]. The spike protein produced by the COVID-19 mRNA vaccine or by SARS-CoV-2 itself induces IL-1β secretion in macrophages [28]. NLRP3 inflammasome is associated with IL-1β secretion. Colchicine has the effect of suppressing the formation of NLRP3 inflammasome [47], which may be useful for vaccine-associated inflammation [48]. The mRNA vaccines increase both immunostimulatory cytokines release and inflammatory cytokines release, especially after the second vaccination and in patients infected by SARS-CoV-2 [10]. The number of deaths after vaccination is higher after the second vaccination (60.6%) than after the first (39.4%) [6]. These similarities may suggest the association in the overproduction of inflammatory cytokines.

### 3.2. Autoimmunity

Another potential cause of post-vaccine adverse reactions is the involvement of autoimmune phenomena [11,12,13,14,15,16,17,18,19]. Autoimmunity may result from IL-6 overproduction and cross-reactivity. IL-6 inhibits regulatory T cell (Treg) differentiation, promotes inflammatory helper T cell (Th17) differentiation, and, thus, induces autoimmune and inflammatory diseases [113]. Autoimmune and inflammatory diseases, including AIH [11,13], AE [60], RA [11], and SLE [99], have been implicated in post-vaccine responses. Autoimmunity may also result from the cross-reactivity between human tissue antigen and the SARS-CoV-2 spike protein [93]. As COVID-19 mRNA vaccines encode the SARS-CoV-2 spike protein, autoantibodies may be produced due to the cross-reactivity between the SARS-CoV-2 spike protein and thyroid protein. A link between vaccine-induced autoimmunity and the human leukocyte antigen gene has also been suggested [88].

### 3.3. Eosinophilia

Eosinophilia may also be responsible for post-vaccine adverse reactions. Eosinophilic myocarditis, eosinophilic pneumonia, EGPA (relapse), eosinophilic cellulitis, eosinophilic panniculitis, and non-episodic angioedema with eosinophilia have been reported following COVID-19 vaccination [20,21,22,23,24,25]. SARS-CoV-1 vaccines have also been shown to induce eosinophilia in the lungs of mice [114]. Andrew et al. reported that the SARS-CoV-1/-2 spike protein might cause eosinophilia associated with a Th2 immune response [115].

### 3.4. ACE2 Downregulation

Post-vaccine adverse reactions may also be the result of the downregulation of ACE2 [26,27]. ACE2 converts angiotensin II (Ang II) to Ang1–7, leading to vasodilation and cardioprotection [26]. The SARS-CoV-2 spike protein in a COVID-19 mRNA vaccine binds to ACE2 and induces ACE2 downregulation. ACE2 downregulation causes an increased level of Ang II and decreases Ang1–7, leading to vasoconstriction and cardiovascular events. The increase in Ang II and the decrease in Ang1–7 trigger the NF-kB pathway, which further promotes the release of inflammatory cytokines, including IL-6, IL-1β, etc. [116]. Myopericarditis is more common in young men than in women, which may be related to the increased level of ACE2 in the latter due to estrogen [117].

## 4. Precautionary Measures Including Exercise, Alcohol Intake, Tobacco Smoking, and Baths

### 4.1. Avoid Strenuous Exercise

High-intensity exercise increases the expression of the NLRP3 gene and inflammatory cytokines (IL-1β and IL-18) compared to moderate-intensity exercise [29]. Moderate-to-low-intensity training is recommended for athletes instead of high-intensity at the time of vaccination [118]. As a precautionary measure against post-vaccination myocarditis in Singapore, young individuals, including children and adolescents, are advised to avoid strenuous physical activities such as running, weightlifting, competitive sports, or playing ball games for two weeks after receiving a COVID-19 vaccination [33]. High school-aged male students tend to exercise more than adult men [119], and because myocarditis occurs predominantly in male adolescents, exercise restriction is recommended. Regarding infection prevention, lymphocytes decrease on the second day following the mRNA vaccination [120], and 3 to 72 h after the vaccination, high-intensity exercise increases the risk of opportunistic infections, according to the open window theory [118].

### 4.2. Avoid Consuming Alcohol and Smoking

Alcohol intake and tobacco smoking cause an increased release of inflammatory cytokines [30,31], coronary spasms [121], and arrhythmia [122]. Alcohol intake and tobacco smoking have also been identified as predictive factors for lower antibody titers after vaccination [123,124]. Alcohol intake increases atrial fibrillation (AF) [122]. On the contrary, limiting alcohol reduces the incidence of AF [122]. Therefore, avoiding alcohol consumption and tobacco smoking is important for increasing antibody titers and preventing adverse reactions, such as coronary spasms and arrhythmia.

### 4.3. Take a Shower Instead of Sitting in a Hot Bath

Taking a bath improves sleep quality, vascular functions, and insulin sensitivity. In contrast, sudden deaths have been associated more frequently with bathing [125]. Inflammatory cytokines, especially IL-6, also increase immediately after bathing [32]. The exact relationship between bathing and COVID-19 vaccination is unknown. However, there have been many sudden deaths while bathing after vaccination in Japan (50 cases; 29 females, 58% and 21 males, 42%; median age 80 (IQR 73–86) years) [34,35] (Figure 1). On the contrary, there have been no reported deaths related to bathing after the influenza vaccine in 2019–2020 [126]. Taking a bath is uniquely customary in Japan. Hot baths are less popular outside of Japan. As such, this issue must be addressed locally. The majority of deaths that occurred while bathing were reported within one week (44/50 cases) after COVID-19 vaccination [34,35] (Figure 1). Based on these reported cases, we suggest that, immediately after COVID-19 vaccination and for several days afterwards, individuals should be advised to take showers rather than baths 

## 5. Discussion

Deaths after COVID-19 vaccination usually occur within several days. According to the US surveillance data, the most common cause of death after vaccination is cardiovascular events, followed by cerebrovascular events [5]. Similarly, the Japanese data have shown that deaths during baths are the most common for several days after mRNA vaccination [34,35] (Figure 1). The COVID-19 vaccines promote inflammatory cytokine release [7,8,9,10], and the overproduction of inflammatory cytokines and thrombosis has been documented in cardiovascular pathology [45].

High-intensity exercise, alcohol intake, tobacco smoking, and taking a bath also increase inflammatory cytokine release [29,30,31,32], which may promote cardiovascular events after vaccination. Based on the current evidence, we recommend refraining from high-intensity exercise, alcohol intake, tobacco smoking, and baths immediately after COVID-19 vaccination and for several days afterwards for the prevention of severe adverse reactions, including death. In Singapore, adolescents and younger persons are advised to avoid strenuous physical activities for two weeks after COVID-19 mRNA vaccination [33]. Most post-vaccination deaths have occurred among the elderly (median age: 76 (IQR 66–86) years) [5]. As such, we propose that high-intensity exercise restriction should be recommended for all individuals after COVID-19 vaccination, regardless of age. Alcohol intake and tobacco smoking interfere with the increase in antibody titers after vaccination. Likewise, corticosteroids and immunosuppressive drugs interfere with vaccine efficacy [123,124]. To increase the vaccine’s effectiveness against COVID-19, we recommend refraining from drinking alcohol and smoking immediately after vaccination.

In patients who take corticosteroid and immunosuppressive medication, there is a high risk of aggravation due to COVID-19 infection [127]. In particular, pre-existing respiratory disorder cases, including MG, may take advantage of the vaccination to avoid COVID-19 pneumonia [66]. On the other hand, although reports are limited, vaccines are also known to relapse autoimmune diseases such as MG, GBS, Graves’ disease, and RA [64,66,89,128]. Therefore, autoimmune diseases require careful observation before and after vaccination.

For patients with a history of COVID-19 infection, vaccination may further increase both inflammatory and immunostimulatory cytokines, including IL-6, compared to patients who have not had the infection [10]. Therefore, receiving a COVID-19 vaccine shortly after COVID-19 infection is likely to cause more pronounced inflammation [10] and autoimmunity due to IL-6 overproduction [113]. In Japan, vaccination is recommended about three months after COVID-19 infection for the healthy population [129].

## 6. Conclusions

After COVID-19 vaccination, inflammatory cytokines, autoimmune involvement, eosinophilia, and the downregulation of ACE2 have been reported in relation to various symptoms and diseases. We should recognize these adverse effects and recommend the following precautions immediately after vaccination: limit strenuous exercise, alcohol intake, tobacco smoking, and taking baths.

## Figures and Tables

**Table 1:** Organ-specific diseases associated with the COVID-19 vaccines.

***1. Cardiovascular diseases***	***6. Skin diseases***
Acute coronary syndrome (ACS)	Alopecia areata (AA)
Aortic dissection (AD)	Bullous pemphigoid
Arrhythmia	COVID arm
Heart failure (HF)	Eosinophilic cellulitis (EC)
Myocarditis/Pericarditis	Eosinophilic panniculitis (EP)
Pulmonary embolism (PE)	Erythema multiforme (EM)
Takotsubo cardiomyopathy (TCM)	Herpes zoster (skin, oral and facial palsy)
Vasospastic angina (VSA)	Leukocytoclastic vasculitis
***2. Respiratory diseases***	Non-episodic angioedema with eosinophilia
Asthma attack	Psoriasis
Diffuse alveolar hemorrhage (DAH)	Pyoderma gangrenosum (PG)
Eosinophilic pneumonia (EP)	Steven-Johnson syndrome (SJS)
Interstitial lung disease (ILD)	Subacute cutaneous lupus erythematosus (SCLE)
Sarcoidosis	Urticaria
***3. Gastroenterological diseases***	***7. Endocrine diseases***
Appendicitis	Graves’ Disease
Autoimmune hepatitis (AIH)	Hypophysitis
Bleeding duodenal ulcer	Hypothyroidism
Intestinal obstruction/perforation	Syndrome of inappropriate antidiuresis (SIADH)
Mesenteric ischemia	Type 1 diabetes mellitus
Pancreatitis	Thyroiditis (painful, silent, subacute)
***4. Renal diseases***	***8. Collagen diseases***
Acute rejection of kidney transplant	Anti-neutrophil cytoplasmic antibody (ANCA)-associated vasculitis
IgA nephropathy	Antiphospholipid syndrome (APS)
IgG4 nephritis	Dermatomyositis (DM)
Membranous nephropathy (MN)	Eosinophilic granulomatosis (EGPA)
Minimal change disease (MCD)	Giant cell arteritis (GCA)
Renal thrombotic microangiopathy	Polymyalgia rheumatica (PMR)
Scleroderma renal crisis	Rheumatoid arthritis (RA)
Vasculitis	Systemic lupus erythematosus (SLE)
***5. Neurological diseaes***	Systemic sclerosis (SSc)
Acute disseminated encephalomyelitis (ADEM)	***9. Hematologic diseases***
Acute hemorrhagic leukoencephalitis (AHEM)	Aplastic anemia (AA)
Acute meningoencephalitis	Acquires hemophilia A (AHA)
Bells’ palsy	Autoimmune hemolytic anemia (AIHA)
Cerebral hemorrhage (CH)	Hemophagocytic lymphohistiocytosis (HLH)
Cerebral infarction (CI)	Immune thrombocytopenia (ITP)
Cerebral venous sinus thrombosis (CVST)	Vaccine-induced immune thrombotic thrombocytopenia (VITT)
Chronic inflammatory demyelinating polyneuropathy (CIDP)	***10. Others***
Guillain–Barré syndrome (GBS)	Abnormal menstrual cycle
Multiple sclerosis (MS)	Anaphylaxis
Myasthenia gravis (MG)	Gout flares
Neuromyelitis optica spectrum disorder (NMOSD)	Lymphadenopathy
Parsonage-Turner syndrome (Neuralgic amyotrophy)	Rhabdomyolysis
Subarachnoid hemorrhage (SAH)	Shoulder injury related to vaccine administration (SIRVA)
Thrombophlebitis	Vogt-Koyanagi-Harada syndrome
Transverse myelitis	

## Data Availability

Japanese COVID-19 vaccine administration dates are available online: https://www.mhlw.go.jp/content/10601000/000928692.pdf (accessed on 26 April 2022, in Japanese), https://www.mhlw.go.jp/content/10601000/000928694.pdf (accessed on 26 April 2022, in Japanese).
